# Confocal imaging capacity on a widefield microscope using a spatial light modulator

**DOI:** 10.1371/journal.pone.0244034

**Published:** 2021-02-16

**Authors:** Yao L. Wang, Noa W. F. Grooms, Sabrina C. Civale, Samuel H. Chung

**Affiliations:** Department of Bioengineering, Northeastern University, Boston, Massachusetts, United States of America; Consiglio Nazionale delle Ricerche Istituto di Elettronica dello Stato Solido: Istituto di Fotonica e Nanotecnologie Consiglio Nazionale delle Ricerche, ITALY

## Abstract

Confocal microscopes can reject out-of-focus and scattered light; however, widefield microscopes are far more common in biological laboratories due to their accessibility and lower cost. We report confocal imaging capacity on a widefield microscope by adding a spatial light modulator (SLM) and utilizing custom illumination and acquisition methods. We discuss our illumination strategy and compare several procedures for postprocessing the acquired image data. We assessed the performance of this system for rejecting out-of-focus light by comparing images taken at 1.4 NA using our widefield microscope, our SLM-enhanced setup, and a commercial confocal microscope. The optical sectioning capability, assessed on thin fluorescent film, was 0.85 ± 0.04 μm for our SLM-enhanced setup and 0.68 ± 0.04 μm for a confocal microscope, while a widefield microscope exhibited no sectioning capability. We demonstrate our setup by imaging the same set of neurons in *C*. *elegans* on widefield, SLM, and confocal microscopes. SLM enhancement greatly reduces background from the cell body, allowing visualization of dim fibers nearby. Our SLM-enhanced setup identified 96% of the dim neuronal fibers seen in confocal images while a widefield microscope only identified 50% of the same fibers. Our microscope add-on represents a very simple (2-component) and inexpensive (<$600) approach to enable widefield microscopes to optically section thick samples.

## Introduction

Widefield epifluorescence microscopes have driven numerous advances in the biological sciences and are ubiquitous in laboratories. Despite their powerful capabilities, broad accessibility, and relatively low cost, widefield microscopes cannot exclude out-of-focus or scattered light. In sparsely-populated or sparsely-labelled samples, this weakness has relatively minor impact: The illumination light is focused onto the plane being observed, so out-of-focus objects are illuminated by a lower intensity of light and in-focus objects are more likely to dominate images. The light from these out-of-focus objects, however, is not excluded. It remains diffusely in the image and interferes with imaging. This weakness has spurred the development of scanning techniques such as confocal microscopy, which can reject both out-of-focus and scattered light [1]. The key component in confocal microscopes is a pinhole in the emission path, which excludes out-of-focus light. Point scanning, in combination with the pinhole, also excludes scattered light as only imaging light from the excited location is included in the final image. Exclusion of unwanted light allows confocal to have significantly better resolution and optical sectioning ability than widefield microscopy. The disadvantages of confocal microscopy are greatly increased complexity, moving parts, requirement of synchronization, and consequently, increased cost.

**Fig 1:**
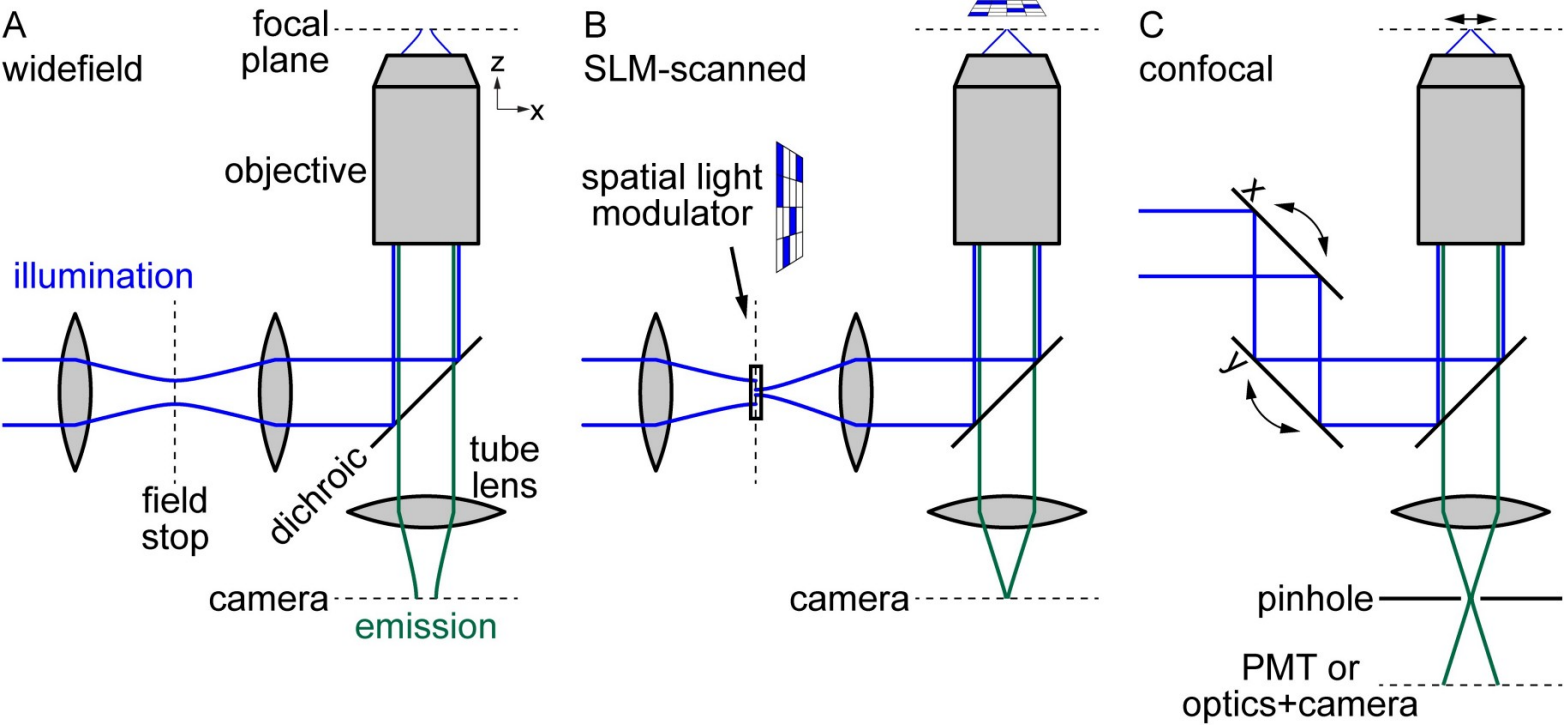
Optics and light beampaths for microscope setups. (A) Widefield illumination optics create field stop plane, whose light distribution is projected to focal plane in sample via objective. Camera images emission light from focal plane. (B) Introduction of SLM at field stop masks illumination light and allows arbitrary illumination patterns at focal plane. (C) Confocal microscope optics scan illumination beam and utilize pinhole to exclude out-of-focus emission light.

The modern widefield epifluorescence microscope (see Fig 1A) is centered around an objective that focuses Köhler illumination light onto a sample and captures emission light from the sample. The emission light is imaged by a tube lens, often onto a camera array. Many widefield microscopes have additional optics in the excitation beampath, represented by two lenses in Fig 1A, that shape and optimize the excitation light. These optics create two planes, the field and aperture (not shown) stops, where masks can be inserted to control the extent and angle of the excitation, respectively.

In contrast to the simplicity of widefield microscopes, confocal microscopes (simplified diagram in Fig 1C) utilize complex sets of optics in both the excitation and emission beampaths. As indicated by the arrows, the excitation optics sweep the excitation light across the sample to image each location in turn. The optics depend on the type of confocal microscope: Laser excitation is typically scanned with mirrors [2]. Spinning disk excitation illuminates multiple, distant points in the sample simultaneously by passing illumination light through a Nipkow disk containing multiple holes [3]. The key optic that distinguishes confocal from widefield microscopy is a pinhole positioned at a conjugate focal plane in the emission beampath. The pinhole strongly filters out light that does not originate from the focal plane, allowing clear imaging of a two-dimensional slice in the bulk without stray light from other depths (*e*.*g*., optical sectioning). Because the emission light comes from different locations in the sample, it can exit the objective at various angles and must be “descanned”. Practically, emission light is typically counterpropagated through the same optics used to scan the excitation beam (not shown in figure for simplicity). It is then spectrally separated by a dichroic mirror and filtered by the pinhole prior to detection by a photomultiplier tube (PMT) for laser scanning confocal or by a camera array for spinning disk confocal.

In our study, we developed simple and inexpensive methods to reduce and exclude scattered and out-of-focus light in a standard widefield epifluorescence microscope. In our approach, we spatially modulate the illumination light and postprocess captured images. Our technique capitalizes on pixelated arrays, such as the spatial light modulator (SLM) and the digital micromirror device (DMD), which modulate the intensity of transmitted and/or reflected light. A transmissive SLM between two crossed polarizers selectively transmits varying intensities of light through each array element. Many projectors use these SLM devices to display an image using incoherent light from a projector bulb. As previously reviewed [4], SLMs and DMDs at the field or aperture stop can control the spatial distribution of the excitation light [5], select the sample location that is observed [6], exclude out-of-focus light [7, 8], and perform structured illumination [9]. Pixelated arrays represent a flexible and cost-effective approach [10] to spatially modulate a light distribution for multiple applications.

Extending the efforts of prior studies, here we show that a transmissive SLM can selectively illuminate locations in the sample with < 500 nm resolution when placed at the field stop of an epifluorescence microscope. We have designed this system for maximum flexibility in illumination spatial pattern and temporal sequence, but, in this study, we simply scan a grid of points across the field of view (FOV), similar to spinning disk confocal. Together with minimal postprocessing, we demonstrate optical sectioning (*i*.*e*., confocal) capability on a typical widefield microscope using an inexpensive SLM add-on. We characterize images taken on our setup and compare them to images from widefield and confocal microscopes. We demonstrate significantly clearer *in vivo* imaging of neurons in *C*. *elegans* compared to typical widefield microscopy. For clarity in our description below, we refer to individual components of the SLM as “elements” and individual components of the camera as “pixels”.

## Results

### Basic optical configuration and concept

In our setup, the SLM is inserted into the field stop position, so that the transmitted light distribution is projected on the focal plane in the sample (see Fig 1B). The illumination originally has a widefield configuration with a large cross-sectional area at the field stop (see Fig 1A). We transmit through isolated SLM elements. The beam transmitting through a single SLM element has a significantly reduced cross-sectional area and is essentially spatially filtered. For simplicity, Fig 1B shows the beam transmitted through a single SLM element to illuminate a single location in the FOV. As described below, we illuminate through many SLM elements, so the true light distribution will comprise many such beams and will change over time. The different beam profiles of the widefield and scanned configurations lead to different intensity profiles in z, which we describe below. In the axial direction, a key functional difference between widefield and SLM-scanned illumination is that the illumination intensity is more strongly peaked at the focal plane in SLM scanning. With an illumination intensity profile more confined to the focal plane, we reduce the emission light from out-of-focus objects and enhance the ability of the microscope to optically section the sample. As described below, we further achieve true confocal operation via postprocessing.

### Implementation

#### Illumination

**Fig 2:**
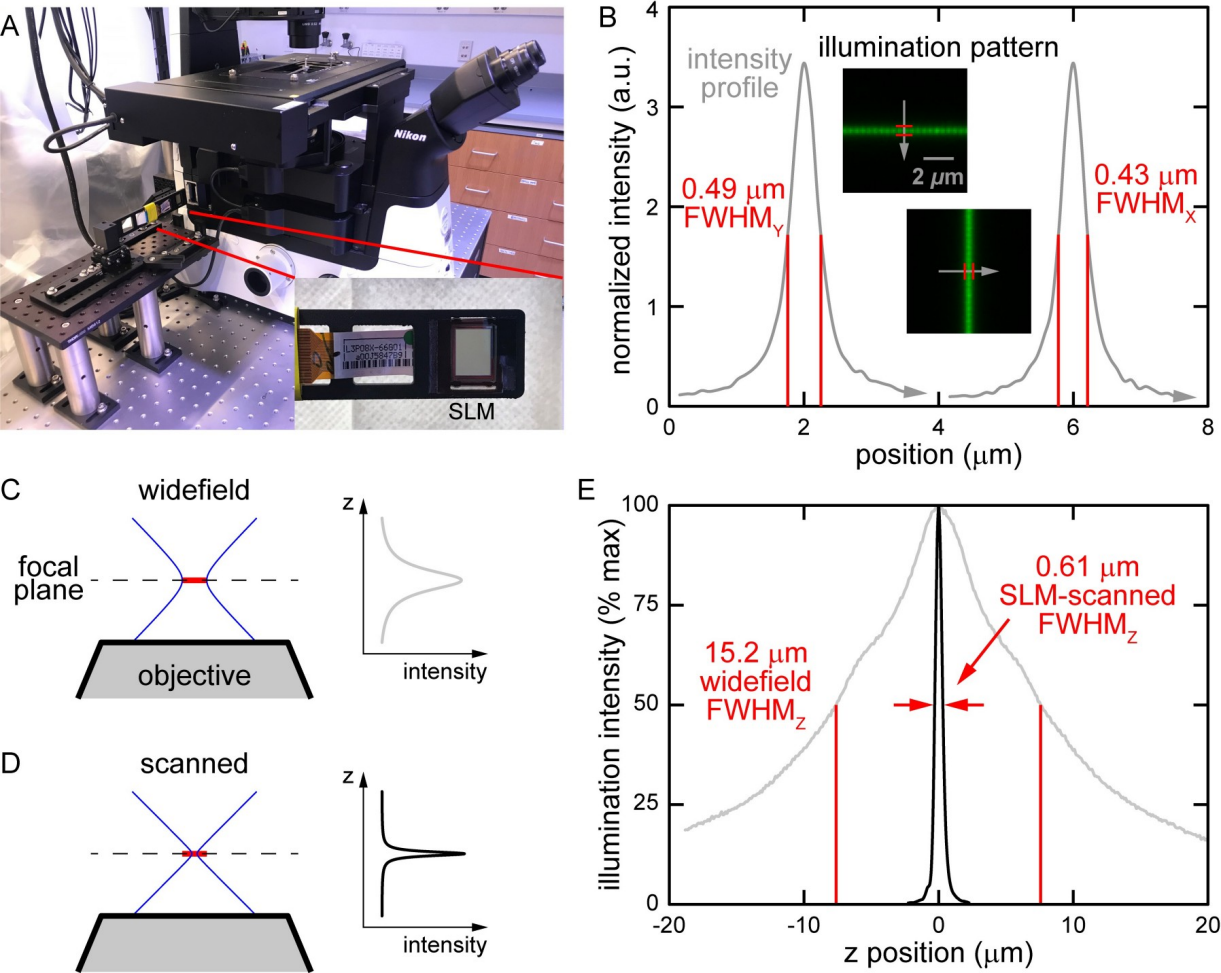
Illumination setup and resolution. (A) SLM on 3D-printed mount on 6-axis fine-positioning stage. SLM inserted into field stop of microscope. (B) Transverse illumination resolution determined using fluorescent dye thin film. (C) Approximate widefield axial illumination beam and intensity profiles. Illumination spans FOV (red). (D) Approximate scanned axial illumination beam and intensity profiles. Beam is scanned across FOV (red line). (E) Measured intensity along optical axis for widefield and SLM-scanned illumination beams. SLM-scanned illumination (black) has significantly better resolution than widefield illumination (gray).

We utilize a transmissive SLM taken from a commercial projector, attached to a 3D-printed mount and a 6-axis stage for precise alignment in the field stop (see Materials and Methods, Fig 2A and S1 Fig). One advantage of using a transmissive SLM is its high accessibility: no other modification to a widefield microscope is required for integration. However, the transmissive SLM has several weaknesses in such implementation. First, its transmission is low, making it challenging to obtain sufficient illumination power, particularly for dim samples, where it could potentially improve imaging the most. Second, the fill factor, or fractional area of the SLM that is actively modulated is low (see S2 Fig), typically slightly above 60%. This low fill factor is due to conduits at the periphery of each element to conduct signals that control the transmission of the active regions. The non-active region casts a shadow at the sample, making the final image pixelated. Even with these weaknesses, we still demonstrate greatly improved imaging by adding a transmissive SLM to our widefield microscope.

We characterized the transverse resolution of the SLM illumination by transmitting only through single lines of SLM elements and illuminating a thin film of fluorescent dye. We measured the illumination resolution, defined by the full width at half maximum (FWHM) as 0.43 μm in the horizontal and 0.49 μm in the vertical directions (see Fig 2B), or an aspect ratio of 0.88. The non-uniform resolution is due to a rectangular active region of our SLM elements. As shown in S2 Fig, we used a brightfield dissecting microscope to measure the aspect ratio of the active region as 11.1 μm to 13.0 μm, or 0.85, producing the difference in x and y resolution observed. We expect a uniform aspect ratio and finer resolution with an improved SLM or a DMD.

We characterized the axial resolution of widefield and SLM illumination using fluorescent beads. As shown in Fig 2C and 2D, widefield and SLM scanned beams have distinct shapes, leading to different properties. A static widefield beam illuminates the entire FOV (red line), unlike a scanned beam, which illuminates a small spot that is moved to sequentially cover the FOV. The beam shapes also produce different axial intensity profiles and different axial illumination resolutions. To measure the intensity profiles, we observe fluorescence of isolated 175-nm fluorescent beads (see Materials and Methods). The beads absorb illumination light of different intensities at specific depths relative to the focal plane. The amount of light the beads emit is roughly proportional to the amount of light they absorb. Thus, by imaging the beads, we obtain a rough measure of the intensity of the illumination light at different depths. As shown in Fig 2E, the widefield illumination intensity profile in z has a wide FWHM of 15.2 μm while SLM illumination through a single element has a narrow FWHM of 0.61 μm.

Conceptually, the axial width of the illumination peak around the focal plane arises from the focusing of the illumination beam. S3A Fig shows the focusing of spatially filtered or collimated light that is propagating parallel to the optical axis. The light focuses to a small spot at the focal plane on the optical axis. S3B Fig shows the focusing of light that is propagating at an angle relative to the optical axis. The light focuses to a similarly small spot at the focal plane, but off the optical axis. As shown in S3C Fig, widefield illumination can be conceptualized as the sum of light beams of many angles (colored gray, blue, black), illuminating entire FOV (red line). Thus, as shown in Fig 2C and 2D, widefield illumination has a wide waist (*i*.*e*., radius of the beam at the focal plane) while scanned beams, including our SLM beams, have a narrow waist. A widefield illumination beam also has a lower effective numerical aperture (NA) than scanned beams. The illumination intensity is I = P / A = P / πr^2^, where I, P, A, and r are the optical intensity, optical power, beam cross-sectional area, and beam radius, respectively. The widefield beam radius more gradually decreases to a wide waist compared to a scanned beam (*i*.*e*., a lower effective NA), so the corresponding illumination intensity peak in z is wide. Conversely, because the scanned beam radius comes to a sharp minimum, its illumination intensity exhibits a sharp peak in z.

**Fig 3:**
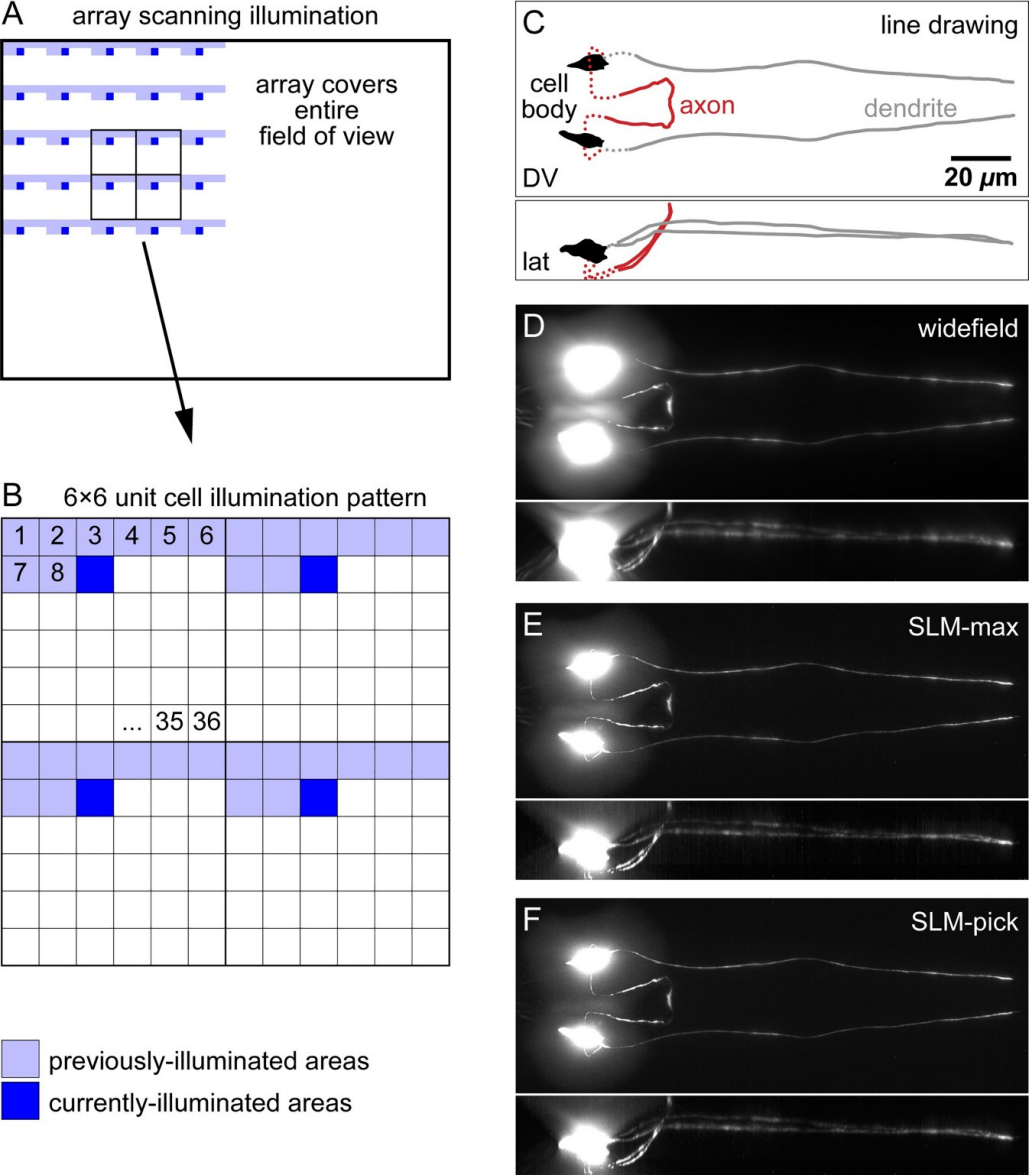
Illumination strategies and postprocessing. (A) Dot array transmitted by SLM illuminates sample. Array is raster scanned across FOV. (B) Expansion of inset in (A). Raster scanning moves single-element illumination through 36 locations of 6 × 6-unit cell. Sub-image of entire FOV is acquired for each of 36 illumination patterns. (C) Line drawing of ASJ neuron imaged. Dorsal-ventral view in xy direction (top), lateral view in xz direction (bottom). (D-F) Fluorescence images, obtained by maximum projections of 3D image. (D) Conventional widefield image. (E) SLM-max image. Pixel value in 3D image is maximum pixel value of 36 subimages. (F) SLM-pick image. Pixel value is in 3D image is pixel value when corresponding sample location is illuminated. Note significantly improved optical sectioning in the max and pick strategies, mostly visibly above and below the cell bodies. Fluorescent image normalization saturates pixels near cell body to visualize dim neuronal fibers.

Stray light arises from fluorescence that does not originate from the sample position under observation, whether inside or outside of the focal plane. Illuminating small areas sequentially and separating these illuminated areas (*i*.*e*., sparse illumination) reduces stray light. We illuminate separated areas in the sample by transmitting a dot array of single SLM elements (see Fig 3A) [8, 11]. Empirically, we determined that separating transmitting elements by five non-transmitting elements (6 × 6-unit cell) eliminates most light interference between illuminated points in our sample (see Fig 3B) while maximizing speed. To illuminate all locations in the FOV, the dot array spans the entire FOV and is raster scanned through all 36 positions in the unit cell. We acquire a sub-image at each position and post-process these 36 sub-images for each final image.

The ideal size of the unit cell is dependent the density of fluorescent structures in the sample and on user requirements for image speed and quality. For instance, a 6 × 6-unit cell illumination scheme requires 36 sub-images while a 10 × 10-unit cell requires 100 sub-images, or about 3× more acquisition time, to cover the same FOV. However, for the reasons stated in the prior paragraph, a larger unit cell will reduce in- and out-of-focus stray light and produce a better image with reduced background. The size of the unit cell is set in the code that controls the SLM.

Because we employed off-the-shelf SLMs, the area in the sample illuminated by a single SLM element is imaged by ~3.5 camera pixels. Along with the non-active region of transmission SLMs, this leads to some minor pixelation and aliasing in the final image (see insets in Fig 2B). We expect to eliminate most of these artifacts by employing reflective DMDs, which have improved specifications. Performance will also greatly improve if the devices are designed for our microscope and camera.

#### Postprocessing

We imaged the ASJ neuron in *C*. *elegans* (see Fig 3C) to test three strategies for postprocessing images. All widefield and SLM-enabled images of *C*. *elegans* were taken with 0.1-s exposure time to facilitate comparison. We compared the resulting images to widefield images taken on the same microscope (see Fig 3D, replicated in S4A Fig). First, we summed the fluorescence values (“SLM-sum”) from the corresponding pixels in the 36 sub-images to calculate the intensity of each pixel in the final image (see S4B Fig). Second, we took a maximum projection of the 36 sub-images, keeping the brightest of the 36 values of each pixel (“SLM-max”) as the intensity of the pixel in the final image (see Fig 3E). In the third (“SLM-pick”), the intensity of the pixel in the final image is the pixel intensity in the sub-image during which the pixel was illuminated by the SLM (see Fig 3F). As expected, the SLM-sum strategy produces an image that is very similar to a widefield image because we capture light while illuminating every position. Light capture occurs sequentially in time rather than simultaneously as it does in widefield. The SLM-max strategy produced a final image that was only slightly inferior to the pick strategy. However, the SLM-max strategy is simpler to implement than the pick strategy for crucial reasons: The SLM-pick strategy requires precise alignment of SLM elements to camera pixels in space and time. Alignment parameters include the vertical location, horizontal location, and rotation around the optical axis as well as tight synchronization of the illumination and observation. Because SLM-sum and SLM-max strategies relax the alignment requirement, they are also significantly more robust to misalignment and instrument drift compared to the SLM-pick strategy. The SLM-max strategy relies on the brightest intensity at a location arising when that location is illuminated. Thus, the resulting image from SLM-max strategy approaches the SLM-pick strategy for sparsely-labelled samples but produces inferior results when bright structures are nearby, (*e*.*g*., in an out-of-focus plane). In addition, the SLM-max strategy retains the brightest pixels and so generally leads to noisier images. The SLM-pick strategy captures fluorescent light from small, separated regions only while they are illuminated. This operation is a “virtual pinhole”, similar to the operation of a physical pinhole in confocal microscopy. Thus, this strategy leads to images with the best rejection of out-of-focus and scattered light. The alignment requirement is readily achievable utilizing a high-precision stage (see Fig 2A), and we use the SLM-pick strategy for the remainder of our study.

### Characterization on well-defined samples

**Fig 4:**
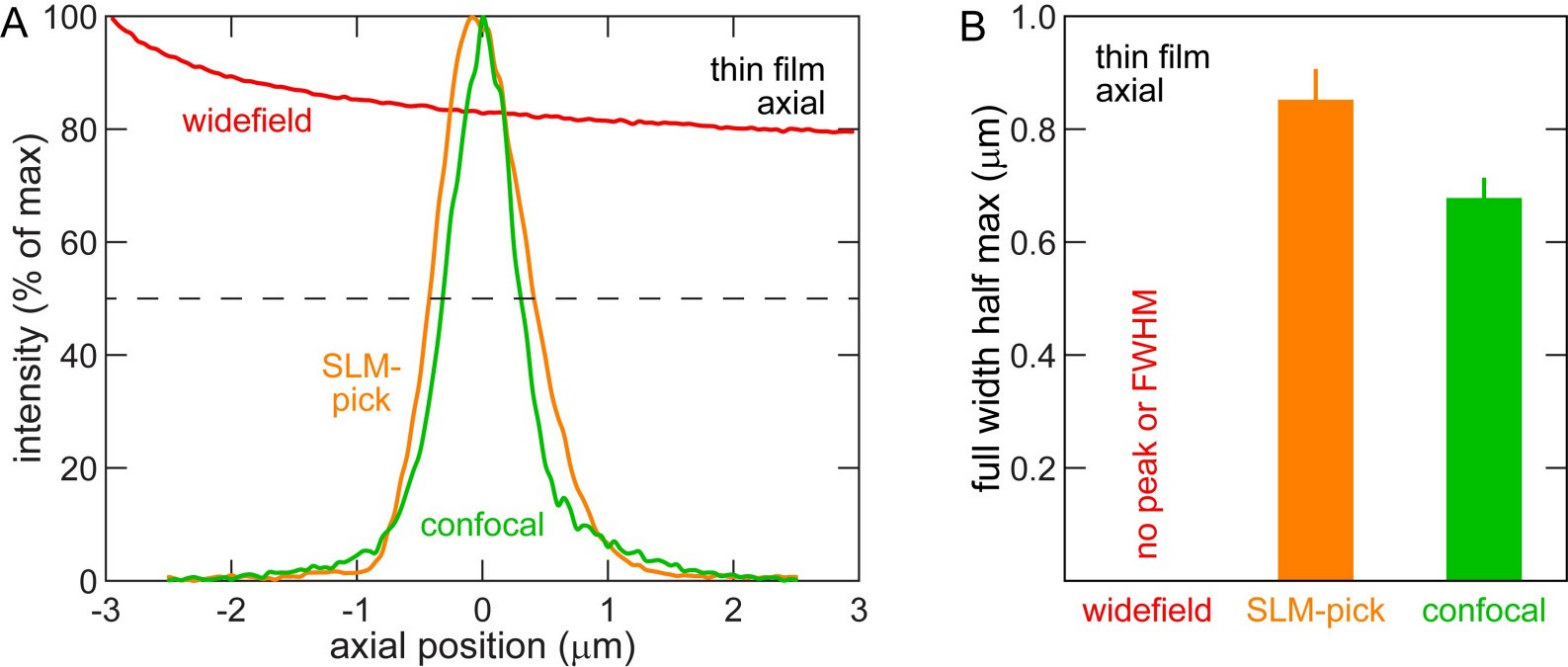
SLM confers sectioning capability of diffuse fluorescent source. (A) Intensity of light at axial positions around fluorescent thin film at *z* = 0. Dotted line at 50% maximum intensity. (B) FWHM of data in (A). *n* = 20 locations. Note optical sectioning capability conferred by SLM.

The axial, or optical sectioning, capability of a fluorescence microscope system can be loosely separated into two regimes depending on the spatial distribution of fluorescent structures: diffuse and localized. We examined the sectioning capability of our SLM-pick strategy compared to widefield and commercial confocal under the two regimes. First, at the extreme end of the diffuse regime is a uniform fluorescence source. We acquired 3D images of a thin fluorescent film. In agreement with prior results [7], axial profiles of widefield microscopy images do not exhibit a measurable peak, indicating minimal sectioning ability (see red curve in Fig 4A). In contrast, the SLM-pick (purple curve) and confocal (blue curve) modalities show sharp peaks with FWHM of 0.85 ± 0.04 μm and 0.68 ± 0.04 μm, respectively (see Fig 4B). The experimental value of the SLM-pick FWHM matches well with a theoretical FWHM value of 0.83 μm, calculated from equation 4 of ref. [12]. This value is calculated with input parameters λ_em_ = 525 nm, *n* = 1.515, NA = 1.4, and PH = object-side pinhole diameter = image-side pinhole / magnification. For our SLM-pick, the effective object-side pinhole diameter corresponds to the area in the sample illuminated by a single SLM element. As stated above, the camera region imaging this area is ~3.5 camera pixels in diameter. Thus, the effective image-side pinhole is 4 camera pixels and PH = 4 pixels * 6.5 μm/pixel / 60x = 0.43 μm. These data indicate that SLM illumination and minimal postprocessing confer optical sectioning of diffusely-labelled samples, as expected from theoretical calculation.

**Fig 5:**
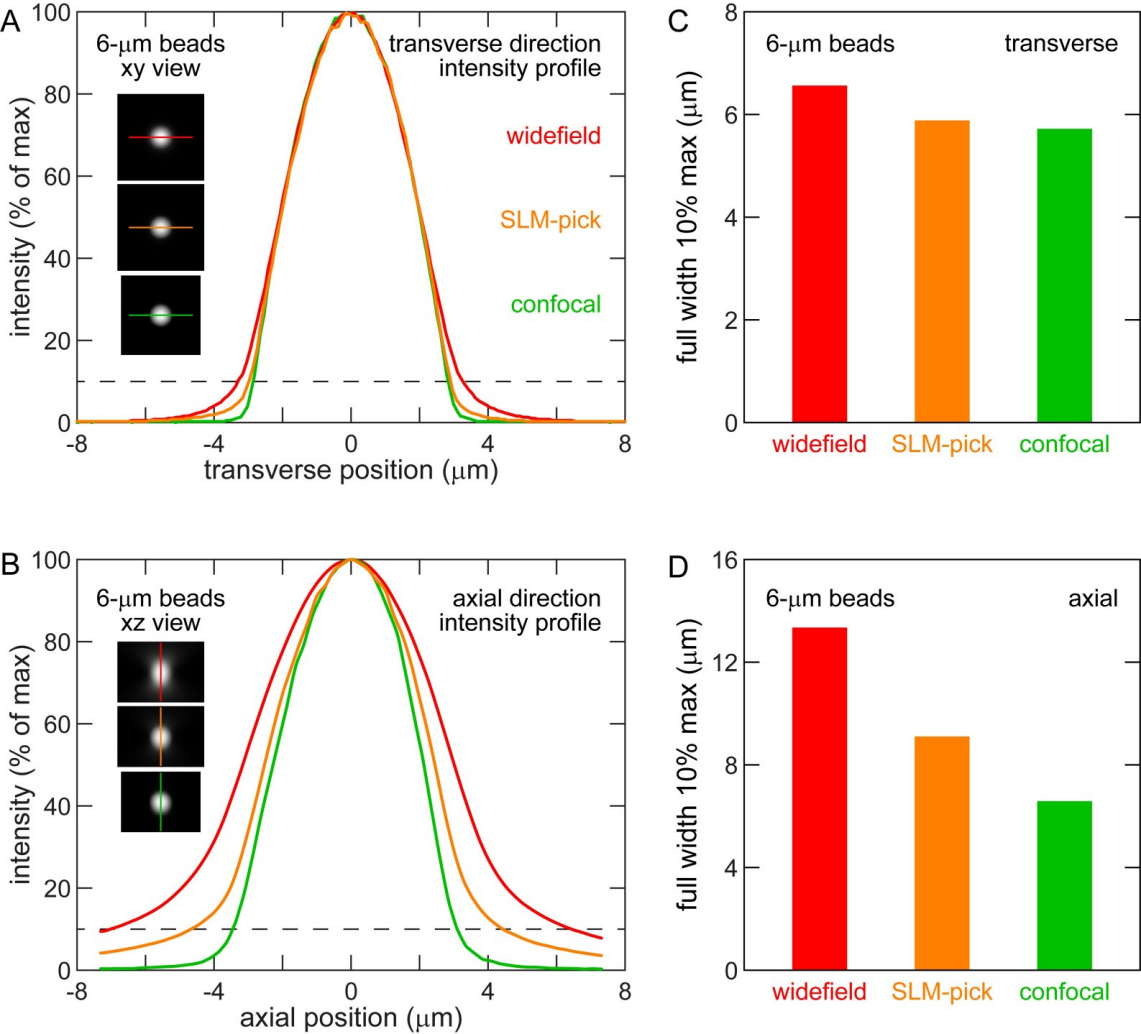
SLM improves sectioning capability of localized fluorescent source. (A) Average transverse intensity profile of 6-μm fluorescent beads. Dotted line at 10% maximum intensity. Insets show maximum projection of average bead images with profile line. (B) Average axial intensity profile of 6-μm fluorescent beads. Insets show maximum projection of average bead images with profile line. (C) Transverse full width at 10% maximum intensity in (A). (D) Axial full width at 10% maximum intensity in (B). Note improved confinement of fluorescence to true bead extent in SLM imaging compared to widefield, particularly in axial direction. *n* = 85 (widefield), 90 (SLM-pick), and 63 (confocal) beads.

Second, we examined the optical sectioning capability for localized fluorescence sources. This regime of optical sectioning is typically of greater interest to microscopists, as fluorescent samples are often composed of sparsely-labelled, localized structures. As mentioned in the introduction, widefield microscopy has some sectioning capability in this regime, just from defocus of light. However, because widefield microscopy does not exclude out-of-focus light, the light from bright out-of-focus objects can impinge on and overwhelm the fluorescence of nearby dim objects of interest. This is true near the bright cell bodies of our neurons, which are 20-30× brighter than their fibers. The neuron in Fig 3D has fluorescences of approximately 20,000 (cell body), 1,000 (axon), and 800 (dendrite) counts. We acquired 3D images of 6-μm fluorescent beads to mimic out-of-focus fluorescence from round cell bodies. Fig 5A shows transverse intensity profiles, and Fig 5B shows axial intensity profiles through the center of an average image of 50 beads. To better highlight the impact bright cell bodies have on dim nearby fibers, we measured the full width at 10% max (FWTM). This value represents the approximate spatial range where objects with 10% the brightness of the bead (see dotted lines) are obscured by stray light from the bead. In the transverse direction (see Fig 5C), the FWTM of the widefield, SLM, and confocal differ by < 15% due to the transverse confinement of focused light. In the axial direction (see Fig 5D), the confocal FWTM is significantly smaller than the widefield FWTM because of improved optical sectioning. The SLM-pick imaging optical sectioning is about 30% greater than confocal but about 50% less than widefield. These data indicate that the addition of an SLM to a widefield microscopy system improves optical sectioning of localized fluorescent sources.

While the SLM-pick strategy improves optical sectioning, we did not observe enhancement of resolution by either the SLM-max or SLM-pick strategies. As shown in S1 Table, the transverse and axial FWHM of sub-diffraction-limit beads is unaffected by use of the SLM. This is because the resolution of our illumination (~0.45 μm) is significantly greater than the diffraction limit (~0.2 μm).

Image acquisition times vary by the imaging method used. For widefield imaging of our beads and *in vivo* samples, exposure times as low as 0.02 s provide sufficient signal. For consistency with SLM images, however, we use the same exposure time for widefield and SLM-scanned imaging. Our SLM has relatively low transmission, leading to measurements that are light-starved. Thus, we use 0.1-s camera exposure time for each sub-image. 36 sub-images result in total 3.6 s total exposure time for an image. Together with the data transfer and postprocessing time—which depends on hardware and software—our overall acquisition time for a 2048 × 2048-pixel 2D image is ~5 s. We expect a significant reduction in acquisition time with an improved SLM and optimized hardware. For the confocal, which raster scans across the sample, the PMT acquisition time for each pixel was 0.38 μs and the total acquisition time was ~4 s.

### Demonstration *in vivo*

**Fig 6:**
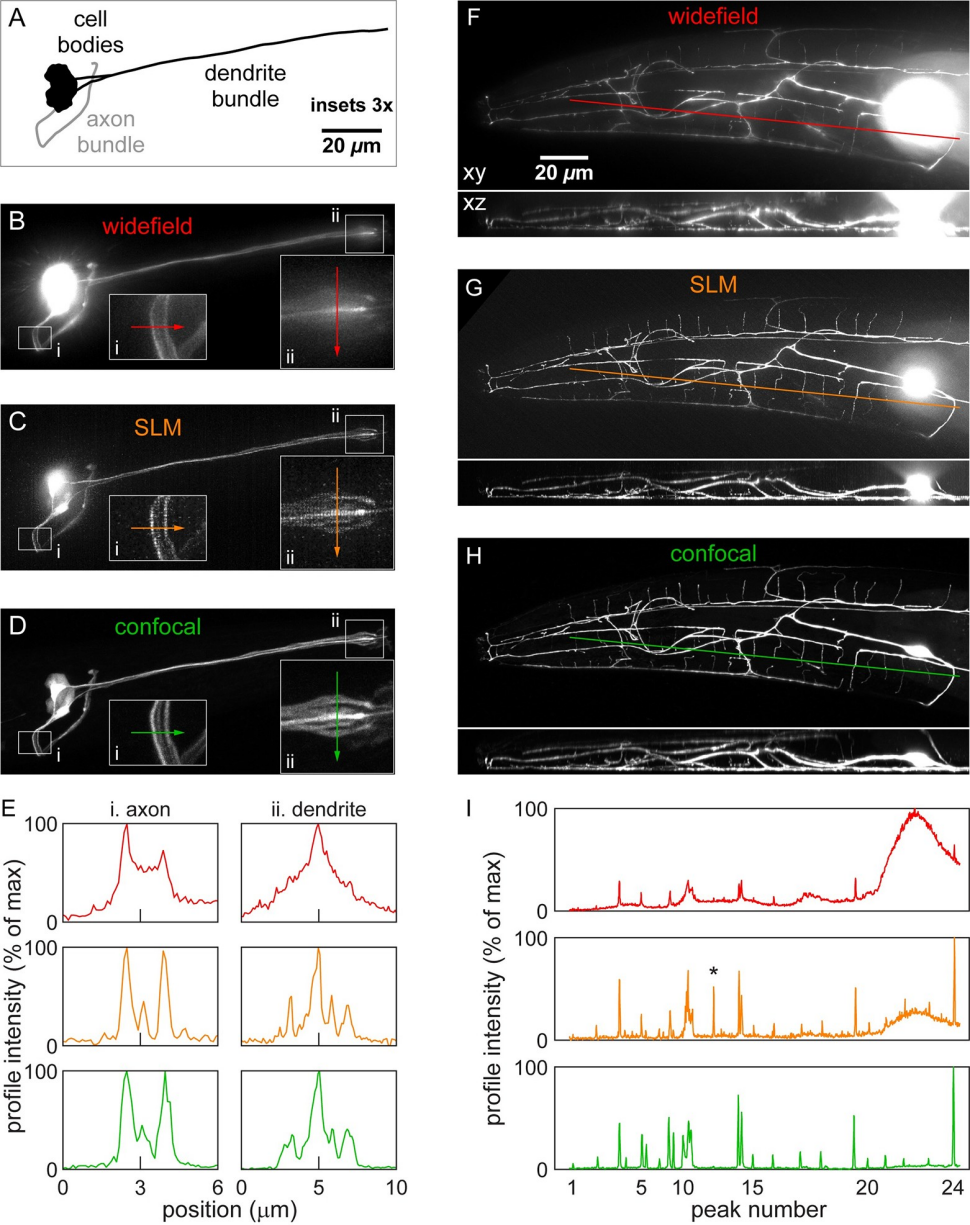
*In vivo* imaging of fluorescent neurons in *C*. *elegans*. (A) Line drawing of amphid neurons imaged. General location of cell bodies and neuronal fibers shown for simplicity. (B-D) Fluorescence amphid neuron images taken by widefield (B), SLM-pick (C), and confocal (D) microscopes. Insets show 3x expanded view of axons (i) and dendrites (ii) with line profiles. (E) Intensity profile of axons (i) and dendrites (ii). Peaks correspond to clear fibers in fluorescence images (B-D). Note correlation of peaks in SLM and confocal profiles and large background obscuring fibers in widefield profile. (F-H) Fluorescence images of FLP neuron taken by widefield (F), SLM-pick (G), and confocal (H) microscopes. (I) Intensity profile in images (F-H). Note correlation of peaks in SLM and confocal profiles and large background in widefield profile, particularly near cell body. Excluded peak in SLM image indicated by *. Fluorescent image normalization saturates pixels near cell body to visualize dim neuronal fibers.

Utilizing the SLM pick strategy, we imaged two types of samples to demonstrate our technique’s capabilities for *in vivo* imaging and compare performance with widefield and confocal imaging. First, to demonstrate enhanced imaging capabilities at high resolution, we imaged a *C*. *elegans* strain with a fluorescently-labelled class of neurons called the amphids, whose neuronal fibers are tightly bundled (see Fig 6A). Widefield imaging has difficulty clearly resolving individual neuronal fibers due to stray light from nearby structures (see Fig 6B and insets). As they reject stray light, SLM-pick (see Fig 6C) and confocal (see Fig 6D) imaging can resolve individual fibers and have a significantly reduced background. Fig 6E quantifies the intensities of the plot profiles in the insets of Fig 6B–6D. In the axon region (see Fig 6Ei) the confocal and SLM-pick profiles both show three peaks, but the widefield profile only shows two peaks. Likewise, in the dendrite region (see Fig 6Eii) the confocal profile (blue) shows four unambiguous peaks corresponding to dendrites near positions 3.3, 5.0, 5.8, and 6.9 μm. The SLM-pick profile (purple) shows the same four peaks with similar relative intensities, but the widefield profile (red) only shows one unambiguous peak. The widefield image also shows a significantly higher background than SLM-pick or confocal images. Near the center dendrite, the background intensity is more than half of the dendrite intensity. In summary, the imaging of tightly-bundled neuronal fibers shows that SLM-pick imaging rejects stray light, improving the contrast of fibers with their backgrounds and allowing them to be clearly discerned, even at submicron resolution.

Second, to demonstrate enhanced imaging capabilities in 3D and at depth, we imaged a *C*. *elegans* FLP neuron, which has a highly branched 3D dendritic structure [13]. As shown in Fig 6F–6H, we used widefield, SLM-pick, and confocal images of the same FLP neuron to generate intensity plot profiles (see Fig 6I) and compute the signal-to-background ratio (SBR) of each fiber (see Table 1). Throughout the extent of the FLP neuron, the SLM-pick image exhibits on average a 2.9× better SBR compared to widefield. In the widefield image, peaks 20–23 are almost entirely hidden by the cell body, whose contribution to the widefield background is greater than the signal of nearby fibers. Defining a clear intensity peak from a fiber as having an SBR > 1.5, the confocal image identifies 24 fibers along the path shown. The SLM-pick image identifies 23 of those fibers, and the widefield image only identifies 12 fibers. (We disregarded a fiber that crosses the plot profile line in the SLM image (indicated by asterisk) but terminates on the profile line in the other two images.) The strong correlation of SLM-pick with confocal imaging demonstrates the capability of SLMs to enhance imaging throughout the 3D bulk of the sample. Also, the SLM and confocal imaging show enhanced optical sectioning ability at all depths compared to widefield imaging (see 3D rotation in S1 Movie). This is evidenced by thinner, better-defined dendrites in the xz axial view, especially at the deeper layers (upper side of xz views).

**Table 1:** Signal-to-background ratios of neuronal fibers in Fig 6F–6H.

peak number	signal-to-background ratio (SBR)			SBR_SLM_/SBR_widefield_	SBR_SLM_/SBR_confocal_
	widefield	SLM	confocal		
1	2.5	3.2	7.0	1.3	0.46
2	2.5	8.6	8.6	3.4	0.99
3	5.3	20.5	36.5	3.9	0.56
4	1.2	2.7	7.2	2.3	0.38
5	3.6	9.4	13.4	2.6	0.70
6	1.3	3.5	29.3	2.8	0.12
7	1.8	4.7	8.7	2.7	0.54
8	4.6	11.0	25.5	2.4	0.43
9	1.3	4.0	40.7	3.0	0.10
10	1.5	6.2	22.7	4.2	0.27
11	1.8	3.4	3.8	1.8	0.88
12	1.7	7.6	11.6	4.4	0.65
13	2.6	15.6	46.0	6.0	0.34
14	2.6	9.4	22.7	3.6	0.41
15	1.4	3.5	11.3	2.6	0.31
16	2.2	5.4	12.7	2.4	0.42
17	1.4	2.8	10.2	2.1	0.27
18	1.2	3.4	25.3	2.9	0.13
19	2.9	10.8	67.0	3.7	0.16
20	1.2	2.8	7.9	2.4	0.35
21	1.0	1.9	8.9	1.9	0.21
22	1.0	1.7	3.5	1.7	0.48
23	1.0	1.5	2.8	1.5	0.53
24	1.3	6.4	26.3	5.0	0.24
average	2.0 ± 1.1	6.2 ± 4.7	19.1 ± 15.9	2.9 ± 1.2	0.42 ± 0.23

SBR is peak pixel intensity corresponding to fiber divided by average of pixel intensities 1–1.5 μm away from peak. Peak number corresponds with labels in Fig 6I. Aggregate calculations are represented as average ± standard deviation.

## Discussion

Our study uniquely combines components and techniques of many prior studies and setups. First, there is an effort to spatially modulate the illumination, as reviewed in [4]. In laser-scanning confocal and two-photon microscopy, the laser beam intensity is controlled by electro- or acousto-optic modulators. In widefield imaging, SLMs or DMDs pattern the illumination and can scan samples without macroscopically-moving parts. Prior studies have shown that modulation of the beam can reduce photobleaching and phototoxicity [14], optically section the sample [8, 15], or increase dynamic range [16] depending on the implementation. Second, there are efforts to replace the pinhole and PMT of confocal setups by an array camera [17–19]. One technique that is very similar to ours is called Image Scanning Microscopy, which rescans the emission beam of a confocal microscope after the pinhole and utilizes an camera array [17, 20]. Several studies use more complicated postprocessing, such as Gaussian masks and deconvolution to achieve confocal capability in widefield imaging [11, 21]. The studies above lay the foundation for our study, which details one of the simplest and most cost-effective methods for converting a widefield into a confocal microscope. The cost of confocal microscopes often renders them inaccessible to individual laboratories. In our setup, a single SLM ($70) mounted onto a high-precision stage ($500) are the only add-ons required to give confocal capabilities to a ubiquitous instrument.

We utilized an SLM to modulate the light distribution at the field stop of our inverted microscope to project an arbitrary illumination pattern on the focal plane in the sample. This opens the door to numerous adaptive or sample-sensitive techniques that can minimize photodamage, increase dynamic range, or improve imaging, such as those mentioned above. These techniques can be especially powerful for sparsely-labelled samples common in fluorescence microscopy. In this study, we show that the SLM-max postprocessing produces images with optical sectioning similar to SLM-pick postprocessing while relaxing requirements for alignment and stability in space and time. This optical sectioning is possible because beam scanning illuminates with high intensity only near the focal plane. Utilizing this setup with SLM-pick postprocessing, we rigorously characterized optical sectioning of well-defined samples, including fluorescent thin film and beads. Compared to widefield imaging, we demonstrate enhanced optical sectioning and improved signal-to-background at ratio high resolution and at depth in fluorescent neurons *in vivo*.

For demonstration purposes, we utilized an inexpensive, off-the-shelf SLM with limited characteristics (*e*.*g*., transmission, fill factor, and element size). As a result, we experienced challenges in obtaining adequate emission light for imaging deep in some samples and observed pixelation in the resulting images. Out-of-focus fluorescence from bright objects, such as the cell body, also remains in our SLM-pick images because our off-the-shelf SLM has larger elements, necessitating a larger virtual pinhole and reduced optical sectioning. Moreover, one fundamental challenge only partially overcome by our technique is light scattering, which reduces illumination light, removes emission light, and creates stray light. While our virtual pinhole removes stray light, it does not mitigate the other two effects. Even so, our images suffice to demonstrate optical sectioning and enhanced imaging. With an improved SLM or by utilizing a DMD, which has superior specifications, we expect improved optical sectioning and resolution with better contrast and deeper imaging.

## Materials and methods

### SLM setup

Following a prior study [10], we removed an SLM from a digital projector (Epson PowerLite 1810p). The SLM was placed between crossed polarizers (Edmund Linear Polarizing Film XP42-18), aligned by hand using a power meter. We measured a 200:1 extinction ratio (transmission of element when on to off). We used a 6-axis stage (Newport, model 9031) and a custom 3D-printed mount to hold and align the SLM. As shown in Fig 2A and S1 Fig, the 6-axis stage was connected to a slider mount positioned along a custom track mounted on an elevated breadboard. This track allowed the positioner to be easily pulled backward out of the microscope or pushed forward into rough position and locked down with screws. After such rough positioning, the SLM was aligned as described below. We used Matlab 2020a (MathWorks) to control transmission through the SLM elements. The code is available at Github (https://github.com/wormneurolab/SLM-confocal).

### SLM alignment

Utilizing the center row and column elements of the SLM, we projected an image of a cross onto a fluorescent thin film. We adjusted the SLM position and orientation using the 6-axis stage so that the cross was in focus and centered on the camera array. This alignment took about a few minutes and was stable for a day.

### Illumination and imaging

As detailed in Fig 3A and 3B, utilizing the MATLAB to control the SLM we illuminated the sample with 36 raster-scanned dot array patterns and captured 36 raw sub-images sequentially. Each sub-image contains information from the full camera array with 1/36^th^ of the FOV illuminated. The exposure time depends on intensity of the light source, transmission of the SLM, strength of fluorescent labelling, and sensitivity of the camera. We used 0.1-s exposure time for widefield and SLM-enabled imaging. Utilizing our setup and samples, we acquired all the raw data for a 2D SLM image in ~ 5 s. This extended time was primarily due to the low transmission of our SLM. We expect significantly reduced exposure time with an improved SLM or a DMD. For confocal, the PMT acquisition time for each pixel was 0.38 μs and the total acquisition time was ~ 4 s.

### Postprocessing

The SLM-sum and SLM-max strategies produce a final image where the pixel values are the sum and the maximum values, respectively, of the corresponding pixels in the 36 sub-images. The SLM-max strategy is commonly known as a maximum projection. In the SLM-pick strategy the values of each pixel in the final image are the values in the sub-image when the pixels are illuminated. Because the pixel-to-element ratio is not an integer (~3.5) some pixels are partially illuminated by two SLM elements. For those pixels, we used the maximum value of the pixels in the sub-images.

### Fluorescent thin film

Using a fluorescent highlighter pen, we drew a 3-mm diameter spot on a slide. We covered the spot with a coverslip and applied pressure, generating a thin film between coverslip and slide. We taped two edges of the thin film slide and allowed it to dry at room temperature for 4 hours.

### Fluorescent bead

For 6-μm beads, we centrifuged 50 μL of 10% bead solution (Invitrogen I14785) for 1 min, removed 25 μL of the supernatant, and vortexed the remaining 25 μL solution for 5 mins. We dropped 5 μL of the concentrated solution to the coverslip center, then added 5 μL to the same location to increase bead density. We dried the coverslip at 37°C for 5 mins. Then we dropped 7 μL of mounting solution to a slide center, flipped over the coverslip, and placed it on the slide without shearing movement. We dried the slide at 37°C for 15 mins and sealed the coverslip edges with wax. For 175-nm beads (Molecular Probes P7220), we followed established procedures [22].

### Microscopy

We used widefield, SLM-enabled, and confocal microscopy to take 3D image stacks (*i*.*e*., z-stack). We utilized a Nikon Ti2-E inverted microscope with a SOLA SE II LED light engine and a 1.4 NA, 60x objective for widefield and SLM-enabled imaging. We utilized a Zeiss LSM 800 microscope with a 1.4 NA, 63x objective for confocal imaging. For thin films, we imaged a 6-μm depth with 50-nm step size. For 6-μm beads, we imaged a 20-μm depth with 250-nm step size. For characterizing imaging resolution using 175-nm beads, we imaged a 6-μm depth with 50-nm step size. We describe animal imaging below. For measuring the intensity profile in the axial direction using 175-nm beads, we imaged a 40-μm (widefield) or a 4.5-μm (SLM-scanned) depth centered on the bead with 100-nm (widefield) or 50-nm (SLM-scanned) step size.

### Image analysis

We averaged thin film and bead data by aligning multiple measurements. For thin film, we used the z position of maximum brightness (found by Gaussian fitting) to align and average profiles together. For 6-μm fluorescent beads, we located each bead and found z position of its center using the ImageJ [23] plugin “3D Objects Counter” (https://imagej.net/3D_Objects_Counter). Using the nearest z slice image, we employed a 2D Gaussian to fit the bead image in x and y. Thus, we obtained the bead’s center position, and utilizing this position, we averaged bead images together and generated transverse and axial profiles. For 175-nm beads, we followed established procedures [22] utilizing the analysis software PSFj [24] to measure the point spread function to assess resolution.

For measuring the intensity profile in the axial direction, we summed pixel intensities within a fixed region of interest. For each depth, we summed all pixel intensities within a 12.96-μm (widefield) or 3.24-μm (SLM-scanned) radius. The beads appear as Airy rings of varying size. This summation radius was chosen to include the largest Airy ring (*i*.*e*., furthest z) and include all bead light captured by the camera. From this sum, we subtracted a summed background fluorescence measurement to yield the bead emission intensity at each depth. This emission intensity is proportional to the illumination intensity.

To calculate the signal-to-background ratio (SBR) of fluorescent fibers in an image, we generated intensity plot profiles along the lines indicated (see Fig6F–6I) showing peaks corresponding to the fibers. The SBR of each fiber is the peak pixel intensity divided by the background around each peak. We calculated the background by averaging the intensities of pixels that are 1–1.5 μm away from the peak pixel in both directions. If another peak was present in one direction, we used the average intensity in the opposite direction only.

### *C*. *elegans* cultivation, immobilization, imaging

We followed established procedure for *C*. *elegans* strains cultivation on Bacto agar plates [25] at 15°C, animal immobilization by sodium azide, and imaging [26]. After immobilization, animals were rotated to a desired orientation [27] under a fluorescence stereomicroscope and then imaged under an inverted microscope.

## Supporting information

S1 Fig Solidworks diagram of SLM mount. SLM is attached to 3D-printed mount on 6-axis stage. These parts are attached to a slider mount that allows easy placement into microscope.
(TIF)

S2 Fig Rectangular active region of SLM. Brightfield image of SLM between two crossed polarizers. Bright areas are rectangular active regions of SLM elements. Measurements of brightfield microscope camera given in micrometers.
(TIF)

S3 Fig Axial dependence of illumination intensity. (A) Focusing of spatially-filtered or collimated light parallel to optical axis prior to objective. Light focuses to small spot on-axis. (B) Focusing of spatially-filtered or collimated light at angle to optical axis prior to objective. Light focuses to small spot off-axis. (C) Widefield illumination comprises light beams (*e*.*g*., gray, blue, black) of many angles, evenly illuminating entire FOV (red).
(TIF)

S4 Fig SLM-sum postprocessing strategy. Fluorescence images obtained by maximum projections of 3D image. (A) Conventional widefield image, replicated from Fig 3D. (B) SLM-sum image. Pixel value in image is sum of pixel values in 36 sub-images.
(TIF)

S1 Table Transverse and axial resolution of widefield, SLM-max, and SLM-pick imaging. FWHM of 175-nm fluorescent beads. SLM-max and SLM-pick do not show improvement of transverse or axial resolution compared to widefield, due to illumination area larger than diffraction limit.
(DOCX)

S1 Movie FLP neuron imaged by widefield, SLM-pick, and confocal microscopy. Movie shows 3D rendering of neuron in Fig 6F–6H.
(MP4)
