# Supplementary figures and images for: Confocal imaging capacity on a widefield microscope using a spatial light modulator
Source: PLoS One. 2021 Feb 16;16(2):e0244034. doi: 10.1371/journal.pone.0244034 (PMC7886194; doi:10.1371/journal.pone.0244034)

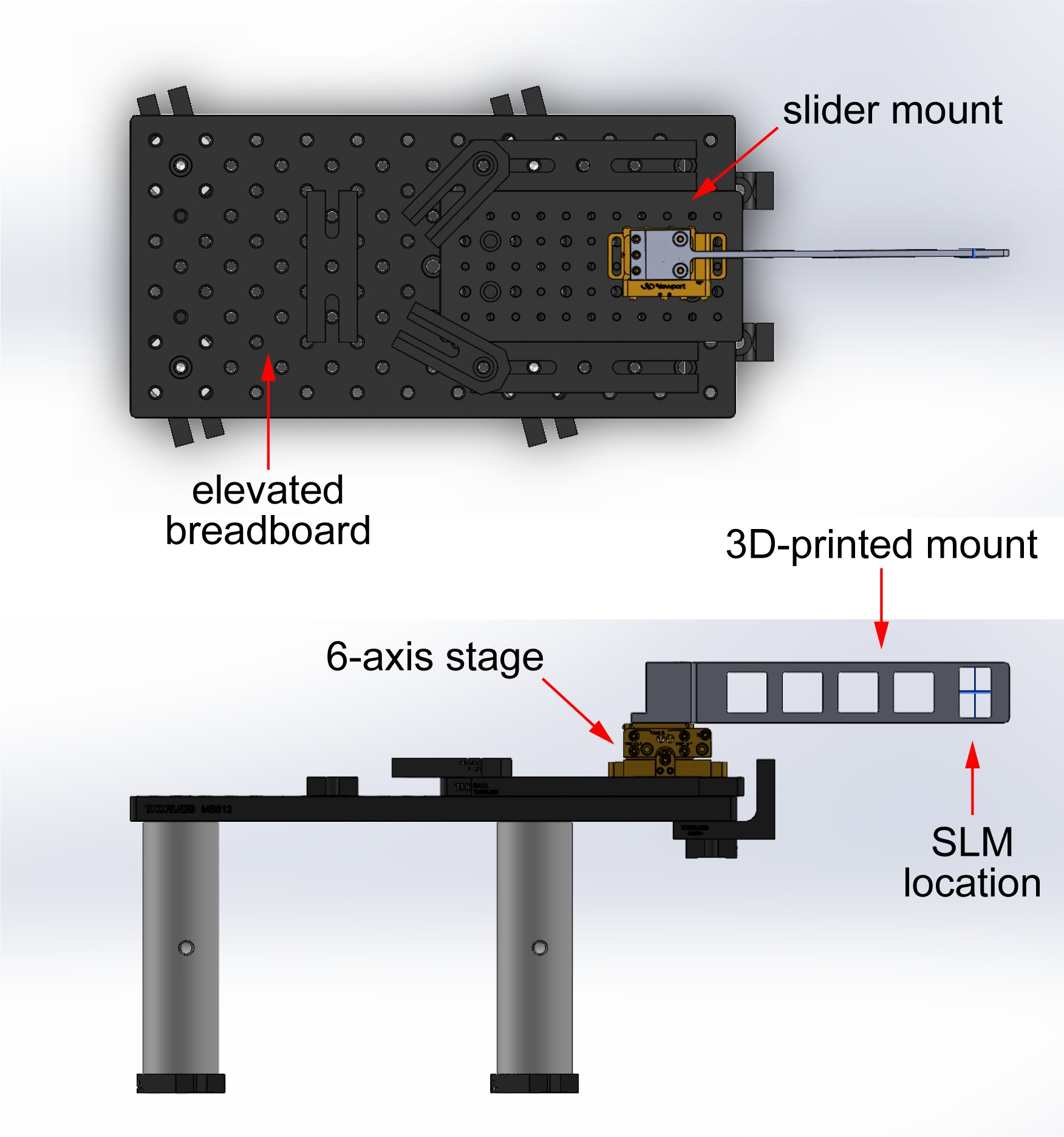

Supplement: S1 Fig — SLM is attached to 3D-printed mount on 6-axis stage. These parts are attached to a slider mount that allows easy placement into microscope. (TIF) [file pone.0244034.s001.tif]

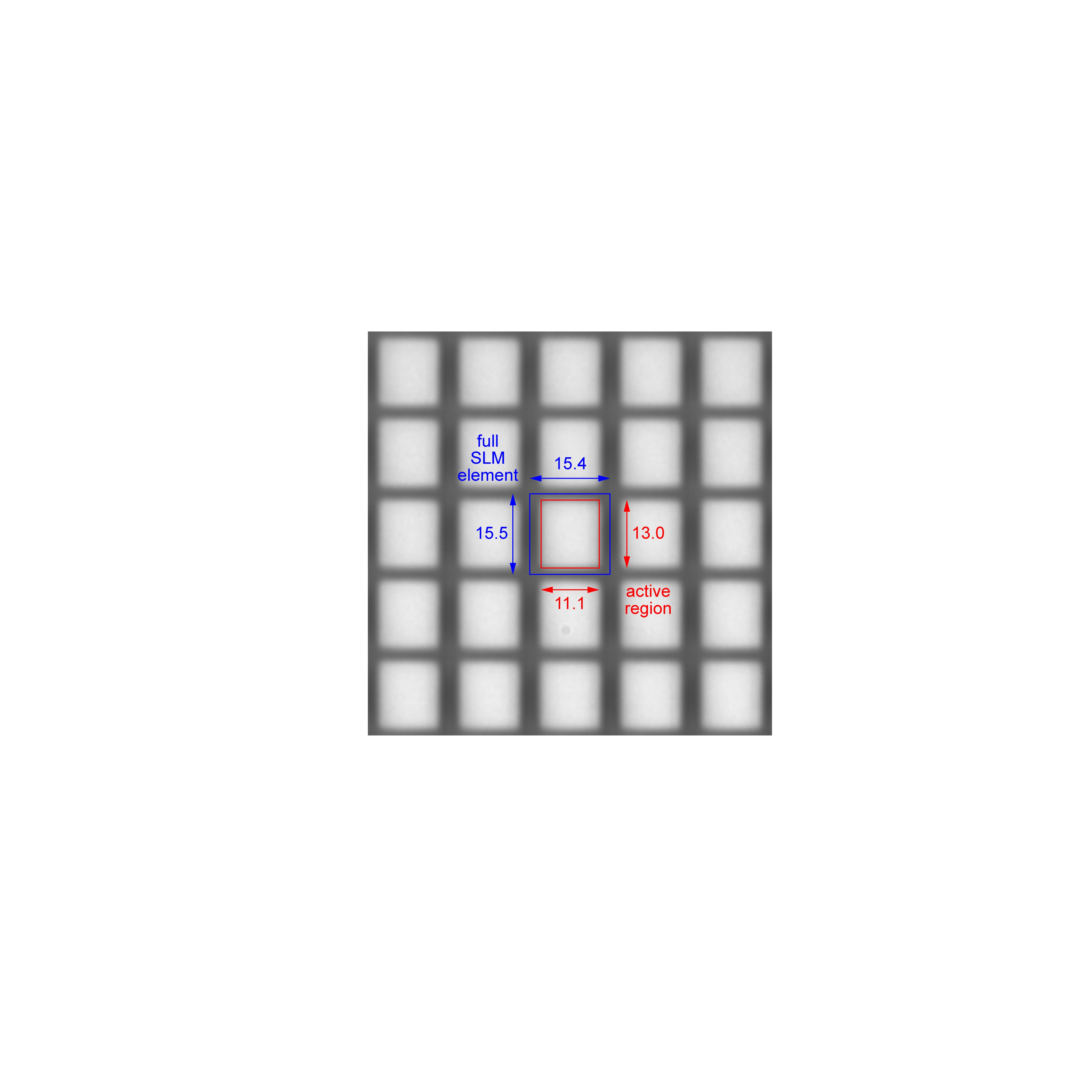

Supplement: S2 Fig — Brightfield image of SLM between two crossed polarizers. Bright areas are rectangular active regions of SLM elements. Measurements of brightfield microscope camera given in micrometers. (TIF) [file pone.0244034.s002.tif]

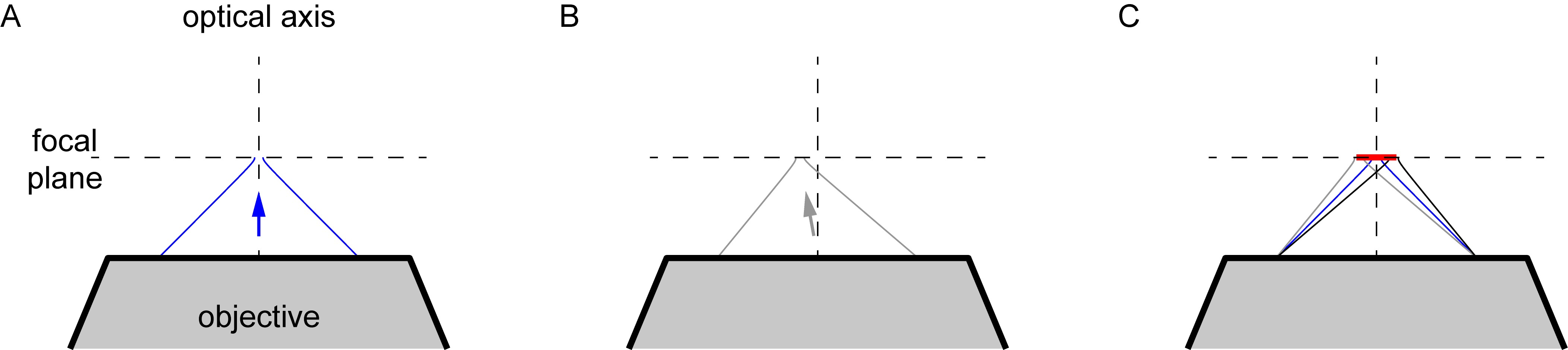

Supplement: S3 Fig — (A) Focusing of spatially-filtered or collimated light parallel to optical axis prior to objective. Light focuses to small spot on-axis. (B) Focusing of spatially-filtered or collimated light at angle to optical axis prior to objective. Light focuses to small spot off-axis. (C) Widefield illumination comprises light beams (e.g., gray, blue, black) of many angles, evenly illuminating entire FOV (red). (TIF) [file pone.0244034.s003.tif]

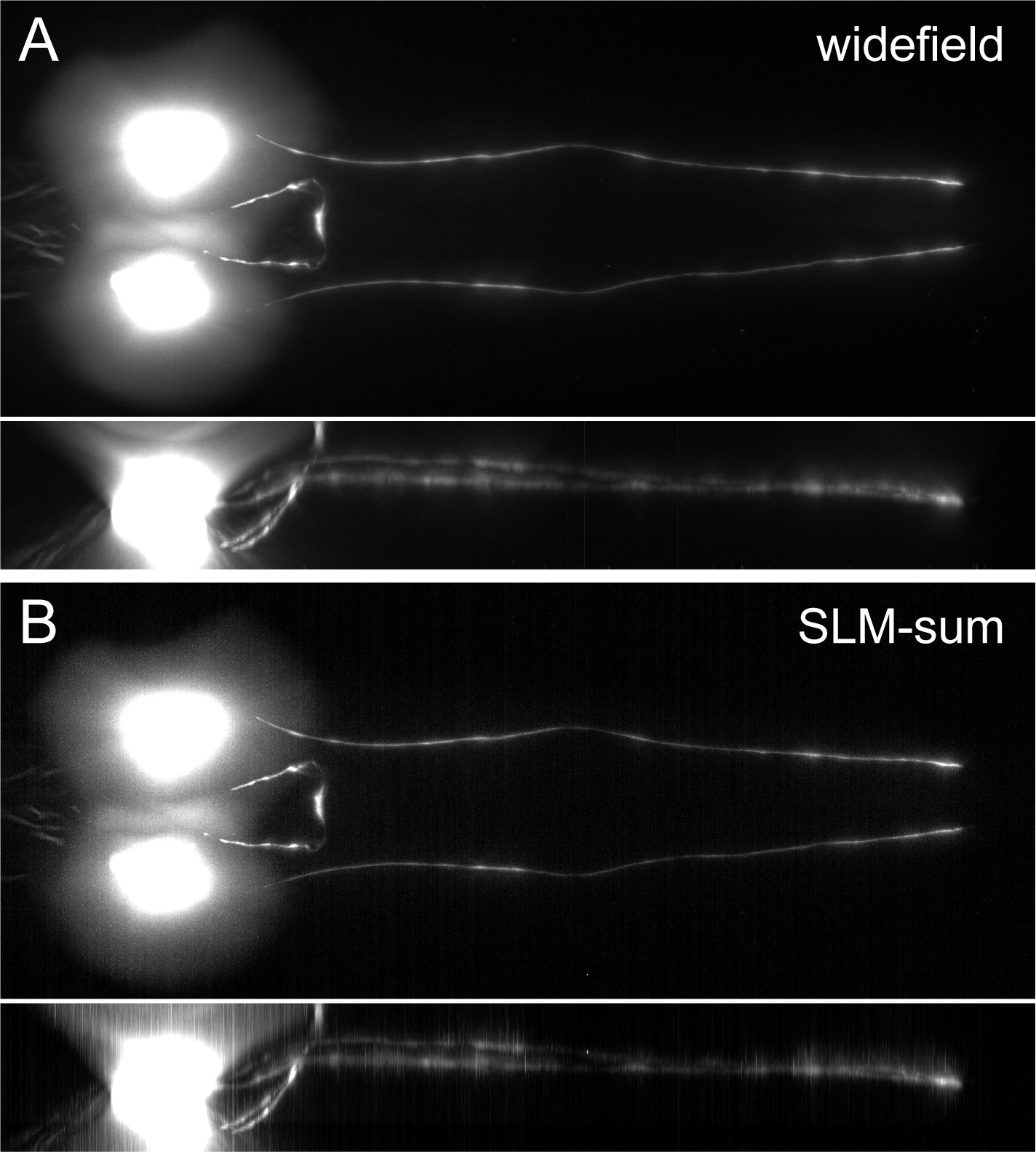

Supplement: S4 Fig — Fluorescence images obtained by maximum projections of 3D image. (A) Conventional widefield image, replicated from Fig 3D. (B) SLM-sum image. Pixel value in image is sum of pixel values in 36 sub-images. (TIF) [file pone.0244034.s004.tif]
