# Supplementary material for: Confocal imaging capacity on a widefield microscope using a spatial light modulator
Source: PLoS One. 2021 Feb 16;16(2):e0244034. doi: 10.1371/journal.pone.0244034 (PMC7886194; doi:10.1371/journal.pone.0244034)
Supplement: S1 Table — FWHM of 175-nm fluorescent beads. SLM-max and SLM-pick do not show improvement of transverse or axial resolution compared to widefield, due to illumination area larger than diffraction limit. (DOCX) [file pone.0244034.s005.docx]

**S1 Table.**

**Transverse and axial resolution of widefield, SLM-max, and SLM-pick imaging.**

|  | widefield | SLM-max | SLM-pick |
| --- | --- | --- | --- |
| FWHM_xy_ | 257 ± 35 | 249 ± 13 | 245 ± 16 |
| FWHM_z_ | 532 ± 58 | 498 ± 79 | 512 ± 90 |
| *n* | 90 | 97 | 48 |
